# Systematic Characterization and Property Enhancement of Recycled PE Flexible Film Packaging

**DOI:** 10.3390/polym17182475

**Published:** 2025-09-13

**Authors:** Johanna Langwieser, Parvin Naderi, Joerg Fischer

**Affiliations:** 1 Competence Center CHASE GmbH, Altenberger Strasse 69, 4040 Linz, Austria; 2 Institute of Polymeric Materials and Testing, Johannes Kepler University Linz, Altenberger Strasse 69, 4040 Linz, Austriajoerg.fischer@jku.at (J.F.); 3 Linz Institute of Technology (LIT) Factory, Johannes Kepler University Linz, Altenberger Strasse 69, 4040 Linz, Austria

**Keywords:** mechanical recycling, flexible film packaging, property enhancement

## Abstract

The production of plastic recyclates is becoming an increasingly feasible and common practice within the industry. Nevertheless, these materials are frequently downcycled for use in applications of reduced value. To bridge the gap between recyclate properties and end-use requirements in flexible packaging, this study investigates the systematic blending of three distinct post-consumer polyethylene (PE) flexible film packaging waste streams with virgin PE materials (low-density and linear low-density). A comprehensive characterization using melt mass-flow rate, oxidation onset temperature, transparency, tensile properties, and puncture properties was conducted on the modified recyclates. Additionally, three commercially available polyethylene-based recyclates and five commercial products containing these recyclates were investigated. The study employs a systematic approach to evaluate the properties of both lab-created and commercial recyclates, as well as those of commercially available flexible film packaging. In light of these findings, recommendations are put forth for the targeted applicability of the recyclates. Results show that key mechanical and optical properties improve with increasing virgin content, though in several cases, up to 80% recyclate content still met or exceeded property requirements for target applications. By enhancing the properties of the recyclate and enabling precise classification for specific applications, this research proposes optimized material formulations and targeted market applications, thereby facilitating the expansion of the scope and value of recycled plastics in the circular economy.

## 1. Introduction

Flexible packaging has grown in importance over the last years. Flexible films are used for the protection of food products, as they delay food spoilage, prolong shelf life, and reduce food waste. For other products, the flexible packaging serves as a barrier to protect against environmental influences and can even adapt to the product’s shape. It can also be used as a means of directly communicating information from the product to the consumer, working cost-effectively by reducing material and enabling convenient storage and transportation [1,2,3,4,5].

Nevertheless, there is not one perfect material that suffices for all the advantages flexible packaging can provide. Flexible film packaging also has different requirements depending on the application, such as sealing properties, tie-layer properties, barrier properties and structural properties. The most commonly used resins in flexible film packaging are polyethylene low-density (PE-LD), polyethylene linear low-density (PE-LLD), ethylene-vinyl acetate (EVA), ethylene-vinyl alcohol (EVOH), polyethylene high-density (PE-HD), polypropylene (PP), polyethylene terephthalate (PET) and polyamide (PA) [1,6,7,8].

The two polymers PE-LD and PE-LLD will be looked at further. PE, in general, has a very simple structure consisting mainly of carbon and hydrogen atoms. Due to this simplicity, PE comes in a multitude of varieties, which are distinguishable by their differences in crystallinity, long and short chain branching, molecular weight distribution, and comonomers [9,10]. PE-LD is a homopolymer and contains long chain branching, which inhibits the ability to form crystals and, therefore, decreases the density. These longer chains provide better processability compared to other PE types and enable better shear-thinning behaviour in the melt. PE-LD exhibits great melt strength, enabling good melt stability in blown film and cast film processes. PE-LD in flexible film packaging is typically used as a sealant or tie resin, as an adhesive or sealant in extrusion coating or as a blending additive in blown film production to improve processing [1,11].

PE-LLD is a copolymer consisting of ethylene and comonomers such as butene, hexene, and octene. Increasing the comonomer content increases the number of short chains, which disrupts the crystallinity and lowers the density. The final properties of the PE-LLD depend strongly on the type of comonomer used. In general, PE-LLD exhibits a higher melting point compared to PE-LD, despite its low level of crystallinity. Due to its structure, it draws down more easily, allowing for thinner layers and is tougher with higher tear strength and impact strength than PE-LD. Typical uses for PE-LLD in flexible film packaging include sealants, components of tie resins, bulking layers, and structural layers. The main difference between PE-LD and PE-LLD is their molecular structure [1,12].

In 2022, in Europe, 7.5 Mt PE-LD and PE-LLD were converted into new plastic products, with their main field of application being the packaging sector [13]. To convert PE-LD and PE-LLD into films, extrusion is the main process. The extruder melts the polymer granulates using a screw and conveys the molten material through a die with special geometry to form the melt into films or coatings [14]. Film formation is typically performed via a blown film process or a cast film process. For the blown film process, the molten material gets pressed through a tubular die and gets rapidly cooled down in a tube form up a tower using air which is forced through the middle of the tube, expanding it in the transverse direction while the whole bubble is pulled in machine direction. Therefore, the produced film is oriented in both machine and transverse directions [15]. Quenching in water is also a possibility, resulting in less orientation and less crystallinity. In the cast film process, the melt is pressed through a flat die, which is then pinned onto a rotating chill roll, which leads to a stretch in machine direction, therefore only orienting in one direction. Other converting processes concerning films include coating or lamination, film orientation, and printing [1].

Due to the different material properties and the different process technologies, the produced films exhibit different properties. A film often needs certain properties such as heat sealing to enable a hermetic seal of the product which needs to be protected, barrier properties against oxygen or moisture, certain strength and/or stiffness to protect against the environment or abuse, adhesion to enable prints or labels, thermoforming, orientation or shrink to mold to the chosen shape or product, and optical properties such as gloss or transparency [1,6,8].

The necessary properties depend on the application of the flexible film packaging and the performance it has to deliver. Food packaging does have different requirements from non-food packaging. However, at the end of their life, all the different types of flexible film packaging meet again during the plastic waste collection process [16,17]. If not handled correctly, flexible film packaging does show some drawbacks as well. Packaging often finds its way into the environment. Pervasive littering results in major environmental issues, including fauna mortality, pollution, obstruction of sewage systems and waterways, and transformation of landscapes. The additional long decomposition period of plastic film packaging further increases the environmental risks [18,19].

When flexible film packaging is collected properly, the end-of-life solutions include landfill, energy recovery, and recycling, differentiating between chemical and mechanical recycling [17]. Chemical recycling destroys materials and breaks down macromolecules into monomers or oligomers that can then be reused, whereas mechanical recycling preserves materials or macromolecules [20].

A conventional mechanical recycling process may include steps such as separation and sorting based on shape, density, size, color, or chemical composition, washing to remove contaminants, grinding to reduce products to flakes, compounding, and pelletizing, and optionally with melt filtration [21]. Each of the steps performed during a recycling process affects the properties of the recycled material [22]. Contaminants may stem from foreign polymers, labels and layers put onto the packaging, or from the product which was packaged into the flexible film. These contaminants can influence the mechanical and optical properties of the recyclate tremendously. Furthermore, the additional conversion process and melt filtration during the mechanical recycling process affect the properties of the recyclate [23]. Therefore, the recyclate and its products made of the used and discarded flexible film packaging often differ strongly from their initial state, inhibiting their usage in the same application field as before [24].

Therefore, it is important to characterize the material properties of the produced recyclates and identify similar or new applications. To broaden the field of applications even more, mixing recyclates with virgin materials is one possibility.

The aim of this study is to systematically identify and characterize the properties of recyclates, including lab-created and commercially available recyclates, as well as the properties of commercially available flexible film packaging. Key objectives include improving certain properties, post-recycling, and enabling precise classification to specific applications. Therefore, three different PE flexible film packaging waste streams are used and converted via a conventional mechanical recycling process on a lab scale. To improve the lab-created recyclates, they are further modified with two different types of virgin material (i.e., PE-LD and PE-LLD) and characterized to identify the properties of the modified materials. Further, three commercially available PE-based recyclates and five commercially available products containing recyclates to different degrees are characterized. Based on the findings, recommendations for optimized material formulations and targeted market applications will be proposed.

## 2. Materials and Methods

### 2.1. Materials

Three distinct plastic film waste streams were selected. The first waste stream originated from the already sorted household lightweight packaging collection, classified as DSD 310 and specified by Duales System Deutschland GmbH (Cologne, Germany). The films in this sorted fraction are larger than DIN A4 and consist of at least 92% polyethylene (PE). Mixtures with this waste stream will subsequently be referred to as “rPE-mix”.

The second waste stream came from a separate collection from the Upper Austrian collection centers, which are operated by O.Ö: The Landes-Abfallverwertungsunternehmen GmbH (LAVU, Wels, Austria). The films in question were initially discarded packaging items belonging to the local population, situated in close proximity to the designated collection center. In order for the films to be collected correctly, they must have a surface area of more than 1 m^2^. The majority of the films in this waste stream originate from the packaging of furniture, construction materials, and agricultural products. The primary polymer identified in this waste stream was polyethylene low-density (PE-LD). Mixtures with this waste stream will subsequently be referred to as “rPE-LD”.

The third waste stream was procured from a grocery store and was initially utilized for the secure transportation of pallets with food products. The principal polymer in this waste stream was polyethylene linear low-density (PE-LLD). For purposes of simplicity, mixtures with this waste stream will subsequently be referred to as “rPE-LLD”. Detailed information concerning the composition of the three waste streams can be found in a previous study by Langwieser et al. [17].

In order to modify the film waste streams, two distinct virgin polyethylene (PE) types were selected. One of the selected polymers was an FA 5224, a PE-LD type produced by Borealis AG (Vienna, Austria) for film extrusion. The intended applications are for general packaging films and pouches. This polymer will further be referred to as “vPE-LD”.

The second type was a Borstar FB4230, produced by Borealis AG (Vienna, Austria). This PE-LLD type is suitable for use in film extrusion. The recommended applications for this material are food packaging, frozen food packaging, general packaging, and pouches. The polymer will further be referred to as “vPE-LLD”.

In order to facilitate a classification of the virgin modified recyclates, a selection of three commercially available recyclates and five commercially available products with varying recyclate content were subjected to characterization. The three commercially available recyclates were produced by Walter Kunststoffe (Gunskirchen, Austria). The three grades are distinguished by their respective sorting, washing, filtering, and coloration processes. The first, designated P-01, is a PE-LD/PE-LLD polymer from post-consumer recycling that has undergone a rigorous sorting, washing, filtering, and light-coloring process [25]. The second, designated P-03, is a dark-colored PE-LD/PE-LLD polymer from post-consumer recycling. It is a very well-sorted, washed, melt-filtered, and gray-greenish polymer with a strong inherent color [26]. The third product (P-05) is a washed, coarse melt-filtered, dark natural-colored PE-LD/PE-LLD polymer from post-consumer recycling [27]. The selected products included toilet paper packagings with 30% and 50% recyclate content (both approximately 50 µm thickness), a yellow bag utilized for the yellow bag collection (approximately 100 µm thickness), composed of 98% recyclate and 2% additives, and two films for construction applications comprising 100% recyclate (approximately 150 µm thickness).

### 2.2. Pre-Treatment

Before converting the chosen waste streams, the film waste needed to be pre-treated. Therefore, each stream underwent the following steps: Each stream was shredded to a flake size of approximately 30 mm using a Micromat 1500 shredder (Lindner-Recyclingtech GmbH, Spittal/Drau, Austria). Then, 2 kg of the material was put into a tank filled with room-temperature water to remove any excess dirt and any contaminants with a density higher than our polyolefin waste (e.g., metal and other polymers like polyethylene terephthalate, PET). After the swim-sink step, the material was put into washing bags and washed inside of a Miele PW818 EL WEK washing machine (Miele GmbH, Wals, Austria) for 15 min at 80 °C with the addition of 875 g sodium hydroxide (NaOH) with a of purity 99%, (Algin, Neustadt-Glewe, Germany). To remove any residual detergent, the material was rinsed for 5 min at a temperature of 25 °C inside the same washing machine. The material was then removed and put into a Binder FED 56 heating oven (Binder GmbH, Tuttlingen, Germany) for 4 h at 60 °C. Since the bulk density of shredded films was too low for the lab-sized compounder, the shredded films were pelletized using a PP200C pellet press (EverTec, Dieburg, Germany) to reach an approximate pellet diameter of 6 mm.

### 2.3. Preparation of the Recyclate-Virgin Mixtures

Before mixing the recyclate material with the virgin material, the pellets were fed through a twin-screw extruder (ZSE 18MAXX, Leistritz AG, Nuremberg, Germany) equipped with a filtration mesh having a hole size of approximately 0.6 mm (mesh 30). The produced granules of rPE-mix, rPE-LD, and rPE-LLD were then combined as a dry mixture in different percentages with vPE-LLD. rPE-LD and rPE-mix were additionally mixed with vPE-LD. After mixing with vPE-LLD, no material of rPE-LLD was left, and no mixtures with vPE-LD were produced. The percentages of the mixtures are shown in Table 1. The mixtures were then compounded using the compounder mentioned above, and it was equipped with a filter with a mesh of 80 (hole size 0.2 mm). To not inhibit the reading flow, the mixtures will further be noted with the used percentages and the abbreviation “r” for recyclate and “v” if only virgin were used (e.g., the mixture of 20% recyclates and 80% virgin will further be called 20% r and 100% virgin material will further be called 100% v).

The material formulations were selected to systematically evaluate the effect of recyclate content in 20% increments from 0% to 100%, reflecting industry-relevant recycling blends. While this study did not implement a formal Design of Experiments (DoE), the structured gradient approach allowed clear observation of trends in mechanical and optical properties. Future work will benefit from applying advanced DoE methodologies to identify optimal formulations with minimized property compromise.

In order to perform transparency measurements as well as tensile and puncture tests, the compounded mixtures were extruded into two films, one with a thickness of approximately 50 µm and the second with a thickness of approximately 150 µm. The commercially available recyclates were extruded into films of a thickness of approximately 50 and 150 µm. For this purpose, an ME30 measuring extruder (OCS Optical Control Systems GmbH, Witten, Germany) equipped with a 150 mm × 1 mm sheet extrusion die and cast film chill rolls was used. The cooling rollers were set at 30 °C, and the take-off speed was varied in order to accommodate the desired thicknesses of the films. The film was maintained at a tensile force of 6 N and rewound with a force of 7 N.

### 2.4. Characterization

#### 2.4.1. Melt Mass-Flow Rate (MFR) Measurements

The MFR measurement was performed in accordance with ISO 1133 [28] using the Aflow (ZwickRoell GmbH and Co. KG, Ulm, Germany). Approximately 3 g of material was tested with test weights of 2.16 kg at 190 °C. The rPE-LD mixtures were tested with a test weight of 5.0 kg at a measuring temperature of 190 °C, due to the viscosity of the rPE-LD being too low to measure with 2.16 kg. After compaction, the material was heated in the measuring cylinder for five minutes, then the measuring process was started, and six samples were taken from the middle strand. The samples were weighed, and each mixture was tested twice.

#### 2.4.2. Oxidation Onset Temperature (OOT) Measurement

The OOT measurement was performed in accordance with ISO 11357-6 [29] using the DSC 4000 (PerkinElmer Inc., Waltham, MA, USA). Approximately 5.0 ± 0.5 mg of material was tested at a heating rate of 10 K/min from 30 °C to 280 °C. Samples were heated continuously in a synthetic air atmosphere until the heat flow curve deviated from the baseline, indicating the onset of an exothermic event and oxidative degradation as indicated by a baseline shift.

#### 2.4.3. Transparency Measurement

Transparency was determined on the produced films through the use of a PerkinElmer Lambda 950 UV-VIS-NIR spectrometer (PerkinElmer Inc., Waltham, MA, USA) in accordance with ISO 13468-1 [30]. The transmittance spectrum was measured across the visible range of 300 to 800 nm. The recorded spectrum was averaged to obtain a single value. This measurement was conducted twice for each mixture.

#### 2.4.4. Tensile Tests

Tensile tests were conducted in accordance with ISO 527-3 [31] using the zwickiLine Z2.5 testing machine (ZwickRoell GmbH und Co. KG, Ulm, Germany), equipped with a load cell with a nominal force of 500 N. Type 5 specimens were tested with a preload of 0.1 MPa and a testing speed of 50 mm/min until failure by fracture occurred. A minimum of five specimens were tested per direction.

#### 2.4.5. Puncture Resistance Tests

Puncture tests were conducted in accordance with the requirements of EN 14477 [32] using the zwickiLine Z2.5 testing machine (ZwickRoell GmbH und Co. KG, Ulm, Germany). The film mixtures were tested at a test speed of 50 mm/min until failure by breakage occurred. A minimum of ten specimens were tested.

## 3. Results and Discussion

### 3.1. MFR Results

As shown in Table 2, the mixtures of rPE-mix and vPE-LD blends have comparable MFR values of approximately 1.1 g/10 min. The rPE-LD mixed with vPE-LD exhibits increasing values from 1.2 g/10 min for 100% v to 3.3 g/10 min for 80% r. The 100% r shows a lower value again at approximately 1.3 g/10 min. Table 3 shows the mixtures with vPE-LLD. The mixtures with rPE-mix show increasing MFR when the recyclate content increases. The rPE-LD blends show rather stable values around 1.3 g/10 min. The rPE-LLD blends also show increasing values as the recyclate content increases. As can be seen in Table 4, the commercially available recyclates show MFR values of 1.1 g/10 min for P-01, 1.4 g/10 min for P-03, and 1.2 g/10 min for P-05.

In general, the observed MFR values align with the typical extrusion processing requirements, suggesting that these mixtures should be suitable for use in film production [1]. The MFR is a measure of the flowability of a material and is dependent upon the molecular structure of a polymer. A higher molar mass, which is indicative of longer linear chains, has a tendency to entangle and thereby diminish the flow capability of the molten material. The MFR of a given polymer is a fixed parameter, dependent on the specific synthesis process employed. The specific processes employed result in the formation of polymers with varying structural characteristics [33]. Moreover, additives are also a function of the polymer synthesis process. At this stage, additives, such as lubricants, can be introduced, which exert a significant influence on the MFR of the polymer [34]. Prior to reaching the consumer, the MFR is predominantly influenced by synthesis and the actions of the polymer producer.

The selected input stream has a significant influence on the MFR of a recyclate. The processing properties of different products vary, and the MFR is a key indicator of flowability [35]. For instance, cast film processing requires an MFR of 2 to 7 g/10 min, whereas blown film processing necessitates an MFR of 0.1 to 5 g/10 min [1,36]. Accordingly, the MFR of the recyclate is contingent upon the depth of sorting at which the input material was sorted. Moreover, the selected waste streams must undergo preliminary treatment, during which specific procedures, such as washing or the removal of residual contaminants, can impact the MFR. The degree and type of contamination can influence the efficiency of removal processes. Nevertheless, the extrusion process is the primary source of degradation within materials due to thermo-oxidative processes, which can result in either a reduction in MFR due to chain branching and crosslinking or an increase in MFR due to chain scission [37,38,39,40]. Contaminants remaining within the recycled material can also affect the MFR, depending on their shape and size [41,42]. The size, shape, and amounts of the particles are dependent on the original use of the packaging, whether fatty products, inorganic products or others were packaged. During post-treatment, further changes to the flowability are possible due to the addition of stabilizers.

As illustrated in Table 2 and Table 3, the combination of recyclate and virgin material results in a mixture with a combined MFR value. Previous studies have investigated the mixing of polymers with varying MFRs and have identified correlations with specific mixing rules since the MFR does not behave linearly [43,44]. Correlations with mixing rules were not investigated further in this work. Overall, during the MFR measurements, the extruded strands showed a smooth surface, no die clogging and no formation of bubbles and fumes, only some odor was noticeable, which indicates good recyclate quality [45,46].

### 3.2. OOT Results

**Figure 1:**
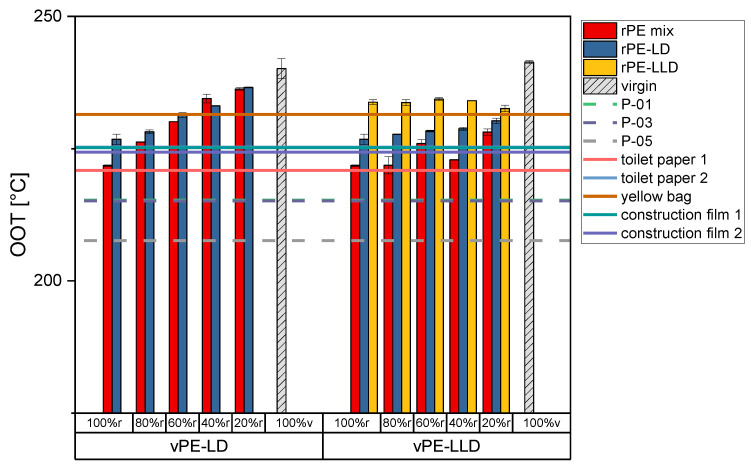
Bar charts of the OOT measurements of the recyclates mixed with virgin PE-LD and PE-LLD in comparison to the commercially available recyclates P-01, P-03, and P-05 and the commercially available products toilet paper 1 with 50% recyclate content, toilet paper 2 with 30% recyclate content, yellow bag with 98% recyclate content and the two construction films made of 100% recyclate, plotted as lines.

Oxidation onset temperature. Figure 1 illustrates the OOT values of the recyclate-virgin mixtures, the commercially available recyclates, as well as the commercially available products. The rPE-mix 100% r exhibits the lowest OOT value, while the vPE-LD 100% v shows the highest value on the left side of the graph. As the proportion of virgin material in the recyclate-virgin mixture increases, the OOT also rises. A comparable trend is observed in the rPE-LD mixtures with vPE-LD.

The OOT values for the recyclate mixtures with the vPE-LLD on the right side of Figure 1 remain rather stable. The rPE-mix values remain at approximately 225 °C, the rPE-LD mixtures at approximately 228 °C, and the rPE-LLD mixtures at approximately 233 °C. The commercially available recyclates all exhibit lower OOT values than those measured for the mixtures. The commercially available products all exhibit values between 220 and 231 °C, with the yellow bag showing the highest value. It should be noted that this product was additionally stabilized during the production process.

As mentioned above, during the synthesis of the polymer and the post-treatment of recyclates, additives are added. While the MFR is influenced by lubricants, the OOT is influenced by antioxidants. The amount of antioxidants used is strongly dependent on the polymer producer and on the field of application of the product made of this polymer. During the lifetime of the chosen product, a certain amount of antioxidants is already used up [47]. Consequently, the consumption of antioxidants results in a reduction in the oxidation temperature, since no protection against oxidation is left. As can be assumed from Figure 1, the virgin materials contain a predetermined quantity of stabilizers, the amount depending on the intended lifetime of the end application [48]. The active stabilizers present in the recycled material are often deteriorated by the time it reaches the end of its useful life [49]. However, small quantities of still active stabilizers may still remain. The incorporation of fresh virgin material thus increases the amount of unused stabilizers, which in turn elevates the oxidation onset temperature. Furthermore, just as the MFR, the OOT is linked to the molecular weight and therefore to the chain length of the polymer, since the polymers degrade due to oxidative degradation [50]. Additionally, the OOT is just as much influenced by contaminants as the MFR. Due to the contaminants containing oxygen in their structure, the recyclate starts to oxidize at lower temperatures compared to the virgin material, which does not contain any contaminants. Nevertheless, all samples, including the 100% r, remain within the typical processing temperatures of PE, ranging between 190 and 250 °C [51]. Consequently, applications that do not necessitate high processing temperatures remain viable with the use of recycled materials, which is in accordance with the measured product samples. The respective degrees of crystallinity can be found in Table S1 in the Supplementary Material. Furthermore, the curves measured using Differential Thermal Analysis (DTA) which were used for the calculation of the OOT and the degrees of crystallinity can be found in Figures S4–S6 in the Supplementary Material.

### 3.3. Transparency Results

**Figure 2:**
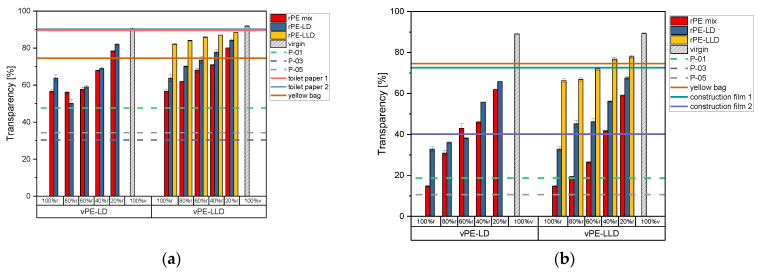
Bar charts of the transparency measurements of the recyclates mixed with vPE-LD and vPE-LLD in comparison to the commercially available recyclates P-01, P-03 and P-05 as well as the commercially available products toilet paper 1 with 50% recyclate content, toilet paper 2 with 30% recyclate content, yellow bag with 98% recyclate content and the two construction films made of 100% recyclate, plotted as lines. (**a**) representing the data of the 50 µm films, (**b**) representing the data of the 150 µm films.

Figure 2 presents the transparency values of the recyclate-virgin mixtures, the commercially available recyclates, and the commercially available products. The lab-created granulates and commercially available recyclates were processed into films with thicknesses of (a) 50 µm and (b) 150 µm, respectively.

On the left side of Figure 2a, the recyclate-virgin mixtures with vPE-LD are illustrated. Notably, the rPE-mix 100% r sample exhibits the lowest transparency value. Upon increasing the proportion of virgin material, a discernible enhancement in transparency is observed at 40% r. As the proportion of virgin material increases further, transparency also rises. Nevertheless, the mixtures remain considerably less transparent than the virgin material. rPE-LD 100% r exhibits higher transparency levels than the mixtures with 80% r and 60% r. At a recyclate content of 40% r, the transparency begins to increase, reaching a maximum at 20% r.

On the right side, Figure 2a illustrates the recyclate-virgin mixtures with vPE-LLD. All three mixtures exhibit a gradual increase in transparency from 100% r content to 100% v content. The lowest values are observed in the rPE-mix samples, while the highest values are evident in the rPE-LLD samples.

Figure 2b exhibits on the left side the transparency values of the 150 µm thick films of the mixtures of rPE-mix with vPE-LD. A steady increase in transparency, accompanied by higher virgin material contents, is noticeable. The lowest value is shown by the rPE-mix 100% r sample. The rPE-LD mixtures with vPE-LD also demonstrate a gradual increase in value with higher proportions of virgin material.

The mixtures created with vPE-LLD on the right side of Figure 2b also demonstrate a consistent upward trend in transparency as the proportion of virgin material is increased. The rPE-mix samples with vPE-LLD exhibit slightly lower values than those with vPE-LD.

Comparing the lab-created recyclates and recyclate-virgin mixtures to the commercially available recyclates, it is noticeable that all mixtures with vPE-LD and all mixtures with vPE-LLD, no matter the thickness, surpass the transparency values of the commercially available recyclates. P-01 exhibits the highest of the three values at approximately 50% transparency, the other two, P-03 and P-05, which lie directly on top of each other in Figure 2b, lie way below P-01.

When comparing the lab-created recyclates and recyclate-virgin mixtures with commercial products, it is important to consider the thickness of the products. At thicknesses of approximately 50 µm, it is noticeable that both toilet paper samples exhibit remarkably high transparency values, which are surpassed only by vPE-LD and vPE-LLD at 100% v content. The transparency values of the yellow bag are less elevated than those observed for the toilet paper packaging. In these instances, the rPE-mix and the rPE-LD blended with vPE-LD at 20% r demonstrate higher values than those observed in the three products. In the case of mixtures comprising vPE-LLD, for mixtures with rPE-mix, only the 20% r exceeds the threshold set by the yellow bag. However, mixtures with rPE-LD 60% r, 40% r, and 20% r are above the threshold. Furthermore, all rPE-LLD mixture samples exceed the threshold.

At a thickness of approximately 150 µm, it is notable that the mixtures with vPE-LD are unable to reach the transparency levels of the yellow bag and construction film 1. It is noteworthy that the rPE-mix at 40% r and 20% r exhibits transparency levels that surpass those of construction film 2, the commercial product with the lowest transparency values. Mixtures of rPE-LD with vPE-LLD demonstrate slightly elevated values in comparison to their counterparts mixed with vPE-LD. However, as was the case previously, they are unable to attain the transparency levels of some of the products. In this instance, the mixtures with 80% r, 60% r, 40% r, and 20% r exceed the transparency value of construction film 2. Mixtures of rPE-LLD with vPE-LLD exhibited the highest values among all mixtures. The rPE-LLD mixtures with 60% r, 40% r, and 20% r recyclate content demonstrated transparency values comparable to those of the yellow bag and construction film 1. All rPE-LLD samples surpassed the transparency values of construction film 2.

The transparency of a film is a property that is dependent on the input stream of the waste, just like for the MFR. Therefore, sorting the material according to colors like transparent, white or colored can strongly influence the transparency of the recyclate. Furthermore, the transparency is influenced by the particles inside the recyclate. These particles can worsen the color of the films, but further, they can lead to an increase in crystallization, or rather, to the nucleation of crystals. Furthermore, crystallization is dependent on the molecular weight and therefore the chain length and chain branching of a polymer, which also influences the MFR and the OOT. Increased chain branching can lead to less crystallization. Chain scission, on the other hand, can lead to increased crystallization. The crystals inside the polymer lead to an increase in internal haze (interior or bulk effects) [1]. Furthermore, the processing method does influence the haze as well, since, as shown in Figure 2, the thickness changes the transparency and since each processing method creates a different kind of surface haze (surface roughness) [1,52]. Additionally, how fast a material is quenched also changes the degree of crystallinity and, therefore, the transparency. Both types of haze, internal and surface haze, are combined, which leads to a value of transparency. Haze and transparency behave inversely to each other; the more haze, the less transparency. The transmission spectra used for the calculation of the transparency values can be found in Figures S7–S9 in the Supplementary Material.

### 3.4. Tensile Test Results

**Figure 3:**
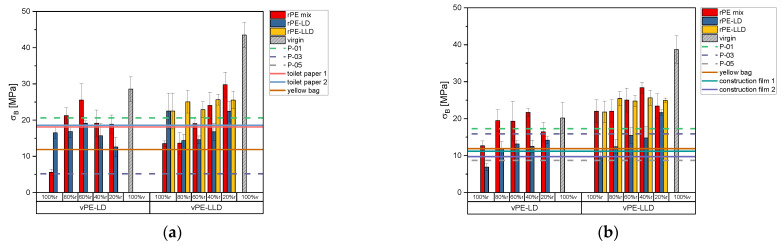
Bar charts of the tensile stress in MD of the recyclates mixed with virgin PE-LD and virgin PE-LLD in comparison to the commercially available recyclates P-01, P-03 and P-05 as well as the commercially available products toilet paper with 50% recyclate content, toilet paper with 30% recyclate content, yellow bag with 98% recyclate content and the two construction films made of 100% recyclate, plotted as lines. (**a**) representing the data of the 50 µm films, (**b**) representing the data of the 150 µm films.

Figure 3 depicts tensile stress data in the MD. For 50 µm films (Figure 3a), 100% rPE-mix samples initially exhibit low stress values, which increase steadily with higher virgin content until 60% rPE-mix with 40% vPE-LD, after which additional virgin material reduces the stress. A similar trend is observed in rPE-LD mixed with vPE-LD. Mixtures with vPE-LLD exhibit varying behaviors: rPE-mix shows increased stress with the addition of virgin material, while rPE-LD initially decreases in stress before rising as virgin content increases. In rPE-LLD with vPE-LLD mixtures, stress increases with increasing virgin material.

Commercially available recyclate P-01 exhibits significantly higher stress values than the lab-created recyclates, surpassed only by rPE-mix 60% r and 80% r with vPE-LD. P-03, with lower stress values than P-01, is exceeded by most recyclate-virgin mixtures. Toilet paper packaging approaches the stress levels of P-01 and is surpassed by selected mixtures (e.g., rPE-mix and rPE-LD with vPE-LD). The yellow bag exhibits intermediate stress values, exceeded by all vPE-LD mixtures and all vPE-LLD mixtures.

For 150 µm films (Figure 3b), a decreasing trend with increasing recyclate content is noticeable for rPE-mix and rPE-LD mixed with vPE-LD. Similar behavior of rPE-mix, rPE-LD and rPE-LLD blended with vPE-LLD is noticeable. Among commercial products, P-01 stress values decrease slightly compared to thinner films, while P-03 values increase but remain lower than P-01. P-05, now measurable due to the higher thickness, displays the lowest values of the three.

When compared to commercial products, only certain mixtures achieve comparable stress values. Most rPE-mix and rPE-LD with vPE-LD, and many rPE-mix and rPE-LLD with vPE-LLD, surpass the stress levels of toilet paper packaging. However, rPE-LD with vPE-LLD matches these values only at 20% r.

**Figure 4:**
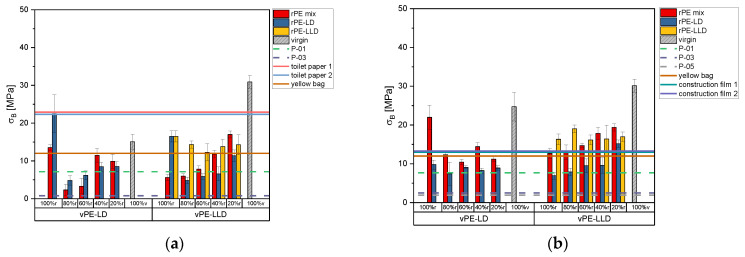
Bar charts of the tensile stress in TD of the recyclates mixed with virgin PE-LD and virgin PE-LLD in comparison to the commercially available recyclates P-01, P-03 and P-05 as well as the commercially available products toilet paper with 50% recyclate content, toilet paper with 30% recyclate content, yellow bag with 98% recyclate content and the two construction films made of 100% recyclate, plotted as lines. (**a**) representing the data of the 50 µm films, (**b**) representing the data of the 150 µm films.

Figure 4 presents the tensile stress data of specimens measured in the transverse direction. For 50 µm films (Figure 4a), the rPE-mix 100% r exhibits a slightly lower value than the vPE-LD, but their mixtures show lower tensile stresses. The rPE-LD 100% r sample exhibits higher tensile stress values compared to the vPE-LD and their mixtures. A sharp decline is observed at 80% r in the mixtures of rPE-mix and rPE-LD with vPE-LD, followed by a gradual increase. Mixtures with vPE-LLD show lower 100% r values than virgin materials, but display a consistent upward trend across all recyclate-virgin mixtures (rPE-mix, rPE-LD). rPE-LLD again shows no clear trend when blended with vPE-LLD.

Commercially available recyclates exhibit relatively low tensile stress values. The P-05 50 µm specimen could not be measured due to preexisting holes, while the P-01 recyclate exhibits similar values to the rPE-mix and rPE-LD mixtures but is surpassed by mixtures containing vPE-LLD. P-03 shows the lowest stress values, exceeded by all lab-created recyclates and virgin-recyclate mixtures. Toilet paper packaging films perform better than the yellow bag but are surpassed by selected mixtures of rPE-mix and rPE-LLD with vPE-LLD.

For 150 µm films (Figure 4b), mixtures of rPE-mix and rPE-LD with vPE-LD exhibit no clear trend, while those with vPE-LLD show a steady increase. Commercially available recyclates have low tensile stress values, surpassed by all recyclate-virgin mixtures. Yellow bag and construction films exhibit the lowest values, surpassed by most mixtures with vPE-LLD and some with vPE-LD.

**Figure 5:**
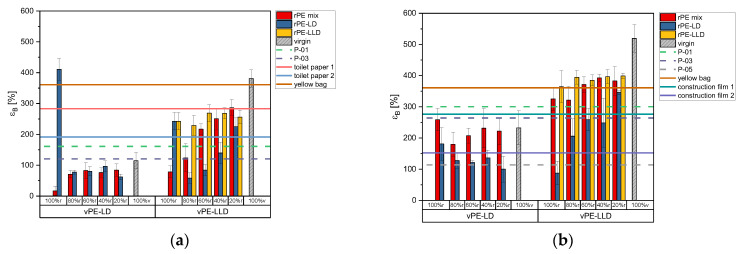
Bar charts of the tensile strain in MD of the recyclates mixed with virgin PE-LD and virgin PE-LLD in comparison to the commercially available recyclates P-01, P-03 and P-05 as well as the commercially available products toilet paper with 50% recyclate content, toilet paper with 30% recyclate content, yellow bag with 98% recyclate content and the two construction films made of 100% recyclate, plotted as lines. (**a**) representing the data of the 50 µm films, (**b**) representing the data of the 150 µm films.

Figure 5a shows the tensile strain of 50 µm films in the MD. The rPE-mix and rPE-LD with vPE-LD exhibit relatively low strain values, but with an increasing trend. Similarly, rPE-mix, rPE-LD and rPE-LLD with vPE-LLD show increasing trends with higher virgin content.

A comparison of the lab-created recyclates with those available on the commercial market reveals that the mixtures with vPE-LD are unable to attain the same values. Conversely, almost all of the mixtures with vPE-LLD exceed these thresholds.

Of the commercially available products, the yellow bag achieves the highest strain, surpassed only by rPE-LLD with vPE-LLD and rPE-mix 20% r with vPE-LLD. Toilet paper with 50% r and construction film 1 have slightly lower values, exceeded by more vPE-LLD mixtures. Toilet paper 30% r and construction film 2 show even lower values, surpassed by a broader range of samples.

For 150 µm films (Figure 5b), strain values are generally higher. The mixture of rPE-mix with vPE-LD shows no clear trend with increasing virgin content, while rPE-LD exhibits a declining trend. Mixtures of rPE-mix and rPE-LD with vPE-LLD exhibit an increasing strain with higher virgin content, but rPE-LLD with vPE-LLD lacks a clear pattern.

Among commercial recyclates, P-01 has the highest strain, exceeded by rPE-LLD with vPE-LLD and rPE-mix mixtures at 60% r, 40% r, and 20% r. Mixtures with vPE-LD fall below P-01 and P-03, while P-05, with the lowest strain, is surpassed by most mixtures with vPE-LD and vPE-LLD.

As shown in Figure 5b, rPE-LLD with vPE-LLD is the only group to exceed the yellow bag’s strain values. Toilet paper 50% r and construction film 1 are surpassed by mixtures of rPE-mix with vPE-LLD, while toilet paper 30% r and construction film 2 are exceeded by all vPE-LLD mixtures and rPE-mix with vPE-LD.

**Figure 6:**
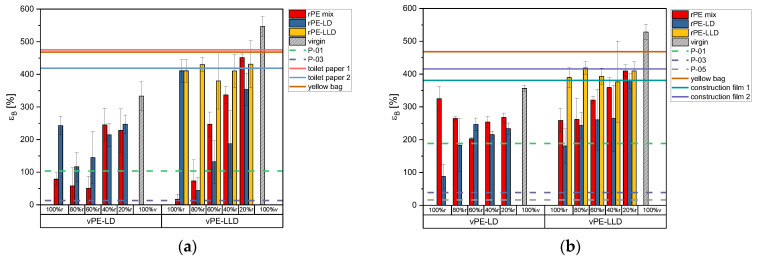
Bar charts of the tensile strain in TD of the recyclates mixed with virgin PE-LD and virgin PE-LLD in comparison to the commercially available recyclates P-01, P-03 and P-05 as well as the commercially available products toilet paper with 50% recyclate content, toilet paper with 30% recyclate content, yellow bag with 98% recyclate content and the two construction films made of 100% recyclate, plotted as lines. (**a**) representing the data of the 50 µm films, (**b**) representing the data of the 150 µm films.

Figure 6a illustrates the tensile strain values obtained for the 50 µm films in TD. It is notable that the tensile strain increases with increasing virgin content in the rPE-mix samples mixed with vPE-LD. A comparable trend is observed for the rPE-LD, albeit with an initial decline from 100% r to 80% r. The rPE-mix combined with vPE-LLD also enhances the tensile strain. A similar trend is evident in the mixtures of rPE-LD with vPE-LLD. Again, no clear trend is noticeable for the rPE-LLD and the vPE-LLD mixtures.

The strain values of the commercially available recyclates remain relatively low. The strain value of P-01 can be surpassed by all mixtures with a recyclate content of 40% or lower. The P-03 reaches only very low strain values. All of the commercially available products reach strain values that are significantly higher than those of the lab-created recyclate films.

As illustrated in Figure 6b, the 150 µm samples exhibit higher strain values than the 50 µm films for some mixtures. However, the trend of increasing strain values with increasing virgin content is consistent with the observations made in previous figures, again with a similar no clear trend for rPE-LLD. The majority of the thicker films demonstrate superior performance compared to commercially available recyclates. Among the commercially available products, only the construction film 1 exhibits higher strain values than some of the rPE-LLD mixtures with vPE-LLD.

Overall, similar to the properties mentioned in this chapter above, the mechanical properties are as well dependent on the input stream, on the molecular weight of a polymer, on the number of contaminants and their size inside the recyclate, and on the processing technique the product is produced with [1,53,54,55]. Concerning the molecular weight, tie molecules and the length of the chains influence the material strongly. As mentioned above, the chain length and its branching influence the degree of crystallinity [56]. The recyclate and the products produced are strongly influenced by the contaminants that are inside. They are defects inside the bulk of the material that lead to premature failure [53].

Furthermore, the processing technique influences the tensile behavior as well, since in the case of films, the thickness makes a big difference, and the orientation of the molecular chains inside the film as well. For cast films, which were produced for this study, a significant difference is noticeable when the film is tested in MD or in TD. The differences in MD and TD can be explained by the processing of the film. In MD, the chains of the polymer are oriented and pre-stretched; since for this study no clamping system or other devices were used to hold the film in shape on the sides, the polymer was able to contract more freely in the transverse direction, so the chains were only pre-oriented in MD [1]. The different states of orientation lead to various failure mechanisms (strain hardening for MD and cold drawing for TD) [2]. The respective stress–strain curves can be found in Figures S1–S3 in the Supplementary Material.

### 3.5. Puncture Resistance Results

**Figure 7:**
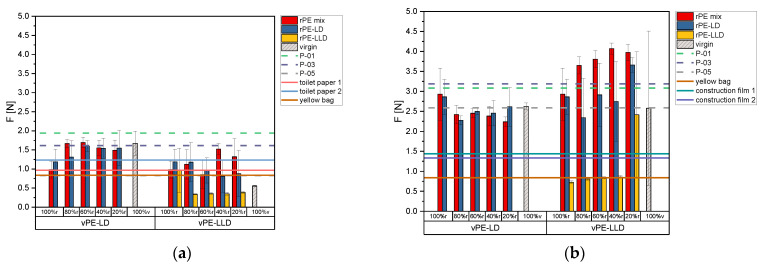
Bar charts of the puncture force of the recyclates mixed with vPE-LD and vPE-LLD in comparison to the commercially available recyclates P-01, P-03 and P-05 as well as the commercially available products toilet paper with 50% recyclate content, toilet paper with 30% recyclate content, yellow bag with 98% recyclate content and the two construction films made of 100% recyclate, plotted as lines. (**a**) representing the data of the 50 µm films, (**b**) representing the data of the 150 µm films.

Figure 7a depicts the maximum force values from the puncture tests for the films with a thickness of 50 µm. For mixtures with vPE-LD, 100% r samples exhibit the lowest puncture forces, with rPE-mix values below 1 N. Adding vPE-LD increases puncture force, reaching values within the standard deviation of 100% v material. The rPE-LD mixtures show higher force values than rPE-mix, though rPE-LD alone exhibits larger standard deviations. Mixtures of rPE-mix and rPE-LD with vPE-LD stabilize after 60% r, while 100% v vPE-LD achieves slightly higher values.

For mixtures with vPE-LLD, 100% r samples exhibit higher forces than the virgin material, though with high variability. The rPE-mix with vPE-LLD shows no clear trend, but values consistently surpass the virgin material. For rPE-LD with vPE-LLD, force values decline as virgin content increases, while 100% r rPE-LLD shows higher force values than its mixtures. Adding virgin material to rPE-LLD reduces force values, which stabilize below those of virgin material.

Commercial recyclates show mixed performance. P-01 achieves the highest force (~2 N), surpassing all virgin and mixture values. P-03 exhibits relatively high values, surpassed only by rPE-mix 80% r and 60% r with vPE-LD. P-05, with lower values, is exceeded by all vPE-LD mixtures and 100% recyclate samples of rPE-mix, rPE-LD, and rPE-LLD.

In regard to commercially available products, the toilet paper 50% r and the yellow bag exhibit comparable values to those observed for the P-05. Toilet paper 50% r and the yellow bag are surpassed by most recyclate-virgin mixtures. Lower-performing products, such as toilet paper 30% r, exhibit values below 1.5 N, outperformed by most rPE and rPE-LD mixtures with vPE-LD. Among vPE-LLD mixtures, only rPE-mix with 40% r and 20% r matches these products.

Figure 7b exhibits the puncture force results of the 150 µm films. Thicker films exhibit significantly higher puncture forces. The 100% r samples of rPE-mix and rPE-LD exhibit higher force values than the 100% v vPE-LD sample. However, adding vPE-LD to rPE-mix decreases force values below those of 100% v material, while rPE-LD mixtures match virgin values but show no clear trend. For mixtures with vPE-LLD, the 100% v sample shows high variability. rPE-mix with vPE-LLD exhibits increasing force values with rising virgin content. However, rPE-LD mixtures show no clear trend, while rPE-LLD values remain lower than those of other materials. Notably, rPE-LLD with 20% r nearly matches virgin material values.

At 150 µm, P-01 and P-03 exhibit high force values, exceeded only by select mixtures, such as rPE-mix with vPE-LLD and rPE-LD 20% r. P-05, with lower force values, is surpassed by a greater number of samples. Most lab-created mixtures, except for rPE-LLD 100% r and its mixtures with vPE-LLD at 80% r, 60% r, and 40% r, outperform the commercially available products.

**Figure 8:**
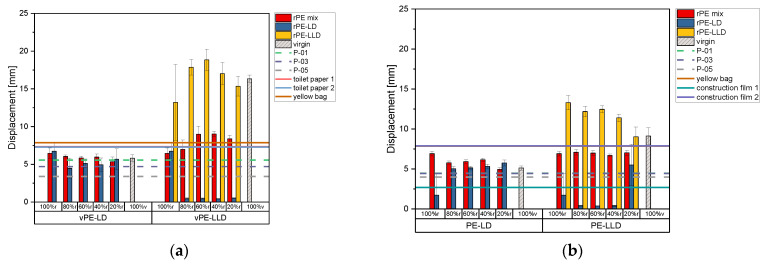
Bar charts of the displacement at maximum puncture force of the recyclates mixed with virgin PE-LD and virgin PE-LLD in comparison to the commercially available recyclates P-01, P-03 and P-05 as well as the commercially available products toilet paper with 50% recyclate content, toilet paper with 30% recyclate content, yellow bag with 98% recyclate content and the two construction films made of 100% recyclate, plotted as lines. (**a**) representing the data of the 50 µm films, (**b**) representing the data of the 150 µm films.

Figure 8a illustrates the displacement values at the maximum puncture force of the 50 µm samples. The 100% r rPE-mix demonstrates slightly higher displacement than the 100% v vPE-LD material, while increasing vPE-LD content results in a gradual decline in displacement for both rPE-mix and rPE-LD. For rPE-LD, 100% recyclate also exceeds virgin material, and displacement decreases with higher virgin content. Mixtures of rPE-mix with vPE-LLD exhibit an upward trend but remain below virgin material values. In contrast, rPE-LD mixtures show a significant decline, even with just 20% virgin content, and remain stable with further additions. The rPE-LLD mixtures show initial increases in displacement at 20% virgin content and again at 60% r but decline with higher virgin content, ultimately falling below 100% virgin material values.

When looking at the commercially available recyclates, P-01 and P-03 exhibit higher displacement values than P-05. Among vPE-LD mixtures, only those containing rPE-mix exceed P-01 values. For vPE-LLD mixtures, those with rPE-mix and rPE-LLD consistently surpass the P-01 threshold. Most mixtures of rPE-mix and rPE-LD with vPE-LD and rPE-mix and rPE-LLD with vPE-LLD outperform P-03 and P-05. However, rPE-LD with vPE-LLD shows significantly lower displacement, falling below all thresholds.

When comparing the recyclate-virgin mixtures to commercially available products, mixtures with vPE-LD do not exceed displacement values of toilet paper packaging or the yellow bag. Only rPE-LLD mixtures, including rPE-mix with 60% r, 40% r, and 20% r, as well as 100% vPE-LLD, surpass these products.

Figure 8b illustrates the displacement value of the 150 µm thick samples. Similarly to the 50 µm samples, rPE-mix with vPE-LD shows a declining trend as virgin content increases, while rPE-LD mixtures exhibit an upward trend. However, rPE-mix with vPE-LD does not reach the displacement value of 100% vPE-LD. The rPE-LD samples display large standard deviations for 100% r and 20% r, with lower variability at intermediate recyclate levels. For rPE-LLD mixed with vPE-LLD, displacement decreases as virgin content rises.

Among commercially available recyclates, P-01 and P-03 display nearly identical displacement values, noticeable by the lines in the figure lying exactly on top of each other, while P-05 exhibits slightly lower values. All rPE-mix samples with vPE-LD exceed the thresholds set by these recyclates. Although the 100% r rPE-LD sample falls below the threshold, its mixtures with vPE-LD surpass it. Similarly, rPE-mix and rPE-LLD with vPE-LLD show significantly higher displacement values than the commercial recyclates, with only rPE-LD mixtures containing 100% r, 80% r, 60% r, and 40% r achieving comparable results. In terms of commercial products, rPE-LLD with vPE-LLD outperforms all of the products. Construction film 1 exhibits values that are surpassed by almost all mixtures except for rPE-LD mixed with vPE-LLD. Yellow bag and constructin film 2 exhibit overlapping values.

The puncture properties are similar to the tensile properties, dependent on the molecular weight, on the additives, and reinforcements which were used beforehand, stemming from the input stream, on the number of contaminants and their size, and the processing methods which were used for the production of the films [57].

### 3.6. Recommendations Based on Virgin-Product Properties

Table 5 presents the property ranges derived from the data sheets of virgin materials designed for specific film products. The typical properties, which were also measured for the purposes of this study, include the MFR, stress and strain at break in both the MD and TD, transparency, and a recommended extrusion temperature. The selected application fields are based on the guidelines provided by Plastics Recyclers Europe [58]. When comparing the laboratory-created recyclate-virgin mixtures to these property ranges, it becomes evident that no single mixture is capable of satisfying all of the property requirements. Nevertheless, certain mixtures have been observed to exhibit specific properties within the desired ranges.

With regard to the potential application of these materials as agricultural films, the mixtures of rPE-mix+vPE-LD (20–80% r) have been found to comply with the MFR and transparency requirements for both 50 µm and 150 µm thicknesses. Similarly, the rPE-mix+vPE-LLD (20–100% r) meets the MFR and transparency ranges for a 50 µm thickness, while fitting a 20–40% r range for a 150 µm thickness. The rPE-LD+vPE-LLD (20–100% r) and rPE-LLD+vPE-LLD (20–100% r) mixtures are also suitable based on the MFR and transparency requirements for both 50 µm and 150 µm thicknesses. However, the mechanical properties remain significantly outside the target ranges concerning the stresses and strains in both MD and TD.

In the context of building and construction applications, mixtures of rPE-mix+vPE-LLD fall within the requisite mechanical property ranges for both film thicknesses. Furthermore, rPE-LLD+vPE-LLD (20–60% r) exhibits matching MFR, mechanical properties, and transparency ranges for both thicknesses as well.

With regard to the production of carrier bags, the rPE-mix+vPE-LLD (40–60% r) is deemed suitable on the basis of its MFR and mechanical properties, concerning both thicknesses. Similarly, the rPE-LLD+vPE-LLD (20–100% r) mixture meets the relevant MFR and mechanical property requirements in MD for both thicknesses, and the transparency level of 20% r is also within the given range for the 50 µm film.

In the context of heavy-duty bags, the mixture rPE-mix+vPE-LLD (20–60% r) is observed to meet the requirements for MFR, mechanical properties in MD for both thicknesses, and partial transparency for both thicknesses. Moreover, rPE-LLD+vPE-LLD (20–100% r) is found to satisfy the MFR, mechanical properties in MD for both thicknesses, and transparency requirements for both thicknesses.

The rPE-mix+vPE-LLD (20–60% r) is deemed suitable for pouch applications with a thickness of 50 µm, based on the assessment of MFR, mechanical properties, and transparency. Conversely, a 20–100% r ratio is recommended for pouches with a 150 µm thickness. Moreover, a 20% r rPE-LD+vPE-LLD mixture exhibits satisfactory matching in terms of MFR, mechanical properties, and transparency. Ultimately, rPE-LLD+vPE-LLD (20–100% r) is found to satisfy the requisite criteria for MFR, mechanical properties in MD for both thicknesses, and transparency for both thicknesses.

In the context of refuse bags, the rPE-mix+vPE-LLD (20–60% r) is observed to align with the MFR and mechanical properties for 50 µm thickness, while exhibiting suitability for 20–100% r based on the mechanical properties for 150 µm thickness. Similarly, rPE-LLD+vPE-LLD (20–60% r) exhibits matching MFR and mechanical properties and demonstrates suitability for 20–100% r based on the mechanical properties for both thicknesses.

With regard to shrink films, the rPE-mix+vPE-LLD (20–60% r) has been demonstrated to satisfy the requisite MFR, mechanical properties, and transparency criteria for a thickness of 50 µm, while also meeting the MFR and mechanical properties requirements for a thickness of 150 µm. Moreover, both rPE-LD+vPE-LLD (20% r) and rPE-LLD+vPE-LLD (20–100% r) are suitable based on MFR, mechanical properties, and transparency. It needs to be noted that conventional shrink film is made of virgin PE-LD [59]. In our study, mixtures with virgin PE-LLD tend to fit better to the chosen properties.

With regard to the stretch film category, the rPE-LD+vPE-LLD (20% r) mixture has been found to satisfy the requisite MFR and mechanical property ranges for both thicknesses. It should be noted that conventional stretch films are made of PE-LLD [60]; however, the category of thin films is outside the scope of this study.

It is crucial to consider the distinction in processing techniques between the original materials and the laboratory-created mixtures, as this can have a significant impact on the properties of the resulting products. The films described in the virgin material data sheets were predominantly produced using the blown film process, whereas the lab-created films were manufactured via the cast film process. Given that the processing method has a significant impact on the mechanical properties, it is essential to consider this difference when evaluating material performance. Nevertheless, all lab-created mixtures can be processed within the recommended extrusion temperature ranges when considering their optimal operating temperatures.

Therefore, suitable blends have been found for every market segment. Modification with virgin considerably expands the spectrum and enables application-specific material development. 

The properties of the commercially available products and the commercially available recyclates can be found in Table S3 in the Supplementary Material.

## 4. Conclusions

This study demonstrates the potential of modified recycled polyethylene (PE) to meet the performance requirements of various flexible film applications. By evaluating key processing and material characteristics, such as melt mass-flow rate (MFR), oxidation onset temperature (OOT), mechanical performance, and transparency, this work systematically identified suitable blends, produced in 20% recyclate content increments and processed into 50 µm and 150 µm films, that are tailored to specific end-use demands.

While some variation in properties was observed among the modified recyclates, the findings emphasize that successful application depends less on matching virgin material properties and more on aligning recyclate performance with the functional requirements of each application. Notably, several formulations containing up to 80% recyclate content were able to meet or exceed property benchmarks for targeted uses. For example, different blends may be optimized for agricultural films, construction films, or packaging pouches, each of which demands a distinct balance of strength, flexibility, and transparency.

In summary, the novelty of this work lies in its comparative analysis of lab-scale recyclate-virgin blends and commercial references, providing data-backed formulations strategies for high-value reuse. This work highlights the importance of a targeted material selection approach when working with recyclates and contributes to the broader goal of enhancing the practical use and sustainability of recycled PE in flexible film packaging.

## Figures and Tables

**Table 1:** Investigated mixtures of waste granulate with virgin PE types.

		Recyclate					
		0%	20%	40%	60%	80%	100%
	100%	X					
	80%		X				
Virgin	60%			X			
	40%				X		
	20%					X	
	0%						X

**Table 2:** MFR measurements of the recyclates mixed with vPE-LD.

		rPE-Mix 2.16 kg		rPE-LD 5 kg	
vPE-LD		MFR	Std.	MFR	Std.
	100% v			1.2	0.01
	20% r	1.1	0.03	1.6	0.06
	40% r	1.0	0.02	2.0	0.04
	60% r	1.0	0.02	2.5	0.04
	80% r	1.1	0.08	3.3	0.05
	100% r	1.1	0.01	1.3	0.02

**Table 3:** MFR measurements of the recyclates mixed with vPE-LLD.

		rPE-mix 2.16 kg		rPE-LD 5 kg		rPE-LLD 2.16 kg	
vPE-LLD		MFR	Std.	MFR	Std.	MFR	Std.
	100% v			0.5	0.00		
	20% r	0.5	0.01	1.5	0.01	0.5	0.01
	40% r	0.6	0.01	1.3	0.01	0.6	0.00
	60% r	0.7	0.01	1.2	0.00	0.7	0.01
	80% r	0.9	0.01	1.3	0.01	0.8	0.01
	100% r	1.1	0.01	1.3	0.02	1.0	0.02

**Table 4:** MFR measurements of the commercially available recyclates.

P-01 2.16 kg		P-03 2.16 kg		P-05 2.16 kg	
MFR	Std.	MFR	Std.	MFR	Std.
1.1	0.01	1.4	0.01	1.2	0.01

**Table 5:** Property ranges of melt flow rate (MFR) stress, strain at break in machine direction (MD) and transverse direction (TD), transparency and recommended extrusion temperature, derived from the data sheets of virgin materials designed for specific film products such as agricultural films, building and construction, carrier bags, heavy duty bags, pouches, refuse bags, shrink films, stretch films, thin films and film recyclates.

	MFR (g/10 min)	Stress at Break (MD) (MPa)	Stress at Break (TD) (MPa)	Strain at Break (MD) (%)	Strain at Break (TD) (%)	Transparency (%)	Recommended Extrusion Temperature (°C)
agricultural films	0.17–1.5	31–62	30–50	470–650	530–750	30.0–92.0	170–220
building and construction	0.3–0.7	12–25	12–23	150–400	410–600	86.0–87.0	170–200
carrier bags	0.25–3.3	22–60	23–25	200–350	500–750	88.0–93.5	185–200
Heavy-duty bags	0.17–4.0	26–62	23–49	200–600	500–770	30.0–92.0	150–230
pouches	0.25–2.1	22–52	20–50	250–650	550–750	30.0–93.0	150–220
refuse bags	0.5–0.7	12–55	12–45	230–570	410–740	30.0	160–190
shrink films	0.3–4.0	22–55	20–50	200–650	500–740	65.0–97.6	150–220
stretch films	1.5–2.0	31–60	20–50	225–650	275–700	92.0–99.3	180–220
thin films	3.3–4.0	20–27	18–22	200–400	500–650	91.0–94.0	150–180

## Data Availability

The original contributions presented in this study are included in the article/Supplementary Material. Further inquiries can be directed to the corresponding author.

## Supplementary Materials

The following supporting information can be downloaded at: https://www.mdpi.com/article/10.3390/polym17182475/s1, Figure S1. Tensile stress-strain curves of (a) rPE-mix + vPE-LD mixtures of 50 µm thickness in TD and MD, (b) rPE-mix + vPE-LD mixtures of 150 µm thickness in TD and MD, (c) rPE-LD + vPE-LD mixtures of 50 µm thickness in TD and MD, and (d) rPE-LD + vPE-LD mixtures of 150 µm thickness in TD and MD. Figure S2. Tensile stress-strain curves of (a) rPE-mix + vPE-LLD mixtures of 50 µm thickness in TD and MD, (b) rPE-mix + vPE-LLD mixtures of 150 µm thickness in TD and MD, (c) rPE-LD + vPE-LLD mixtures of 50 µm thickness in TD and MD, (d) rPE-LD + vPE-LLD mixtures of 150 µm thickness in TD and MD, (e) rPE-LLD + vPE-LLD mixtures of 50 µm thickness in TD and MD, and (f) rPE-LLD + vPE-LLD mixtures of 150 µm thickness in TD and MD. Figure S3. Tensile stress-strain curves of (a) of the commercially available products in TD and MD, (b) commercially available recyclates of 50 µm in TD and MD, and (c) commercially available recyclates of 150 µm in TD and MD. Figure S4. Heat flow curves of (a) rPE-mix + vPE-LD and (b) rPE-LD + vPE-LD. Figure S5. Heat flow curves of (a) rPE-mix + vPE-LLD, (b) rPE-LD + vPE-LLD, and (c) rPE-LLD + vPE-LLD. Figure S6. Heat flow curves of (a) the commercially available products and (b) the commercially available recyclates. Figure S7. UV-Vis spectra of (a) rPE-mix + vPE-LD of 50 µm, (b) rPE-mix + vPE-LD of 150 µm, (c) rPE-LD + vPE-LD of 50 µm, and (d) rPE-LD + vPE-LD of 150 µm. Figure S8. UV-Vis spectra of (a) rPE-mix + vPE-LLD of 50 µm, (b) rPE-mix + vPE-LLD of 150 µm, (c) rPE-LD + vPE-LLD of 50 µm, (d) rPE-LD + vPE-LLD of 150 µm, (e) rPE-LLD + vPE-LLD of 50 µm, and (f) rPE-LLD + vPE-LLD of 150 µm. Figure S9. UV-Vis spectra of (a) the commercially available products, (b) the commercially available recyclates of 50 µm, and (c) the commercially available recyclates of 150 µm. Table S1. Degrees of crystallinities of the lab-mixtures. Table S2. Degrees of crystallinities of the commercially available products and the commercially available recyclates. Table S3. Properties of the commercially available products and the commercially available recy-clates.

## References

[1] Morris B.A. (2017). The Science and Technology of Flexible Packaging.

[2] Barlow C.Y., Morgan D.C. (2013). Polymer film packaging for food: An environmental assessment. Resour. Conserv. Recycl..

[3] FPA—Flexible Packaging Association Advantages of Flexible Packaging. https://www.flexpack.org/advantages.

[4] Sawant S. The Pros and Cons of Flexible Packaging vs. Rigid Packaging. https://www.linkedin.com/pulse/pros-cons-flexible-packaging-vs-rigid-dr-sharayu-sawant/?trackingId=x4sv2eTBTqGXGiHjU5Dkeg%3D%3D.

[5] Ebnesajjad S. (2013). Plastic Films in Food Packaging.

[6] Wagner J.R. (2016). Multilayer Flexible Packaging.

[7] Niaounakis M. (2019). Recycling of Flexible Plastic Packaging.

[8] Bamps B., Buntinx M., Peeters R. (2023). Seal materials in flexible plastic food packaging: A review. Packag. Technol. Sci..

[9] Baur E., Brinkmann S., Osswald T., Rudolph N., Schmachtenberg E. (2013). Saechtling Kunststoff Taschenbuch.

[10] Eyerer P., Elsner P., Hirth T. (2005). Die Kunststoffe und ihre Eigenschaften.

[11] Gedde U.W., Mattozzi A., Albertsson A.-C. (2004). Polyethylene Morphology. Advances in Polymer Science, Long Term Properties of Polyolefins.

[12] Kissin Y.V. (2001). Polyethylene, Linear Low Density. Kirk-Othmer Encyclopedia of Chemical Technology.

[13] Plastics Europe AISBL (2024). The Circular Economy for Plastics: A European Analysis.

[14] Wagner J.R., Mount E.M., Giles H.F. (2014). Extrusion: The Definitive Processing Guide and Handbook.

[15] Cantor K. (2019). Blown Film Extrusion.

[16] van Eygen E., Laner D., Fellner J. (2018). Circular economy of plastic packaging: Current practice and perspectives in Austria. Waste Manag..

[17] Langwieser J., Zeilerbauer L., Fischer J. (2024). Identification of the recyclable content of polyethylene films in the Upper Austrian waste streams. Int. J. Sustain. Eng..

[18] Jalil M.A., Mian M.N., Rahman M.K. (2013). Using Plastic Bags and Its Damaging Impact on Environment and Agriculture: An Alternative Proposal. Int. J. Learn. Dev..

[19] Ujeh K.C. The Negative Environmental Effects of Plastic Shopping Bags. https://www.ibanet.org/article/76F8D2A9-1A1D-4A2F-8A6F-0A70149FD4D5.

[20] Martens H., Goldmann D. (2016). Recyclingtechnik.

[21] Turner R.P., Kelly C.A., Fox R., Hopkins B. (2018). Re-Formative Polymer Composites from Plastic Waste: Novel Infrastructural Product Application. Recycling.

[22] Ragaert K., Delva L., van Geem K. (2017). Mechanical and chemical recycling of solid plastic waste. Waste Manag..

[23] Langwieser J., Fischer J. (2024). Investigation of the Impact of Single and Double Filtration Systems on Post-Consumer PE Film Waste. Polymers.

[24] Bashirgonbadi A., Lase I.S., Delva L., van Geem K.M., de Meester S., Ragaert K. (2022). Quality evaluation and economic assessment of an improved mechanical recycling process for post-consumer flexible plastics. Waste Manag..

[25] Walter Kunststoffe GmbH Produktdatenblatt P-01. http://www.walter-kunststoffe.com/content/de/produkte-regenerate.

[26] Walter Kunststoffe GmbH Produktdatenblatt P-03. http://www.walter-kunststoffe.com/content/de/produkte-regenerate.

[27] Walter Kunststoffe GmbH Produktdatenblatt P-05. http://www.walter-kunststoffe.com/content/de/produkte-regenerate.

[28] (2022). Plastics—Determination of the Melt Mass-Flow Rate (MFR) and Melt Volume-Flow Rate (MVR) of Thermoplastics.

[29] (2018). Plastics—Differential Scanning Calorimetry (DSC)—Part 6: Determination of Oxidation Induction Time.

[30] (2019). Plastics—Determination of the Total Luminous Transmittance of Transparent Materials.

[31] (2018). Plastics—Determination of Tensile Properties—Part 3: Test Conditions for Films and Sheets.

[32] (2004). Packaging—Flexible Packaging Material—Determination of Puncture Resistance—Test Methods.

[33] Cestari S.P., Martin P.J., Hanna P.R., Kearns M.P., Mendes L.C., Millar B. (2021). Use of virgin/recycled polyethylene blends in rotational moulding. J. Polym. Eng..

[34] Samyn P., Schoukens G. (2008). On the efficiency of internal lubricants for polymers under different sliding conditions. Vinyl Addit. Technol..

[35] Akhras M.H., Fischer J. Influence of color sorting on the property profile of polyethylene recyclates. Proceedings of the 38th International Conference of the Polymer Processing Society (PPS-38).

[36] Asaclean Understanding Melt Flow Rate (MFR) in Plastic Processing. https://www.asaclean.com/blog/understanding-melt-flow-rate-in-plastic-processing.

[37] Petukhova E.S., Fedorov A.L., Argunova A.G. (2023). Investigation of Mechanisms of Polyethylene Degradation under the Action of Natural Climatic Factors. Polym. Sci. Ser. B.

[38] Pinheiro L.A., Chinelatto M.A., Canevarolo S.V. (2004). The role of chain scission and chain branching in high density polyethylene during thermo-mechanical degradation. Polym. Degrad. Stab..

[39] Mendes A.A., Cunha A.M., Bernardo C.A. (2011). Study of the degradation mechanisms of polyethylene during reprocessing. Polym. Degrad. Stab..

[40] Langwieser J., Schweighuber A., Felgel-Farnholz A., Marschik C., Buchberger W., Fischer J. (2022). Determination of the Influence of Multiple Closed Recycling Loops on the Property Profile of Different Polyolefins. Polymers.

[41] Yang J., Liang J.Z., Tang C.Y. (2009). Studies on melt flow properties during capillary extrusion of PP/Al(OH)_3_/Mg(OH)_2_ flame retardant composites. Polym. Test..

[42] Koca H.D., Doganay S., Turgut A., Tavman I.H., Saidur R., Mahbubul I.M. (2018). Effect of particle size on the viscosity of nanofluids: A review. Renew. Sustain. Energy Rev..

[43] Cho K., Lee B.H., Hwang K.-M., Lee H., Choe S. (1998). Rheological and mechanical properties in polyethylene blends. Polym. Eng. Sci..

[44] van Krevelen D.W., Nijenhuis K.T. (2009). Properties of Polymers: Their Correlation with Chemical Structure; Their Numerical Estimation and Prediction from Additive Group Contributions.

[45] Gall M., Freudenthaler P.J., Fischer J., Lang R.W. (2021). Characterization of Composition and Structure-Property Relationships of Commercial Post-Consumer Polyethylene and Polypropylene Recyclates. Polymers.

[46] Gall M., Wiener M., de Oliveira C.C., Lang R.W., Hansen E.G. (2020). Building a circular plastics economy with informal waste pickers: Recyclate quality, business model, and societal impacts. Resour. Conserv. Recycl..

[47] Kirschweng B., Tátraaljai D., Földes E., Pukánszky B. (2017). Natural antioxidants as stabilizers for polymers. Polym. Degrad. Stab..

[48] Gijsman P., Kutz M. (2017). Polymer Stabilization. Applied Plastics Engineering Handbook.

[49] AMargolin L., Vorontsov N.V., Monakhova T.V., Popov A.A. (2024). Thermal oxidation of blends of polypropylene and polyamide 6/66. Effect of inhibition. Polym. Degrad. Stab..

[50] Celina M.C. (2013). Review of polymer oxidation and its relationship with materials performance and lifetime prediction. Polym. Degrad. Stab..

[51] Baur E., Harsch G., Moneke M. (2019). Werkstoff-Führer Kunststoffe.

[52] Stehling F.C., Speed C.S., Westerman L. (1981). Causes of haze of low-density polyethylene blown films. Macromolecules.

[53] Traxler I., Fellner K., Fischer J. (2023). Influence of Macroscopic Contaminations on Mechanical Properties of Model and Post-Consumer Polypropylene Recyclates. https://www.researchgate.net/publication/369031050_Influence_of_Macroscopic_Contaminations_on_Mechanical_Properties_of_Model_and_Post-Consumer_Polypropylene_Recyclates.

[54] Long C., Dong Z., Liu X., Yu F., Shang Y., Wang K., Feng S., Hou X., He C., Chen Z.-R. (2022). Simultaneous enhancement in processability and mechanical properties of polyethylenes via tuning the molecular weight distribution from unimodal to bimodal shape. Polymer.

[55] Alzerreca M., Paris M., Boyron O., Orditz D., Louarn G., Correc O. (2015). Mechanical properties and molecular structures of virgin and recycled HDPE polymers used in gravity sewer systems. Polym. Test..

[56] Balani K., Verma V., Agarwal A., Narayan R. (2014). Physical, Thermal, and Mechanical Properties of Polymers. Biosurfaces.

[57] Lange J., Mokdad H., Wysery Y. (2002). Understanding Puncture Resistance and Perforation Behavior of Packaging Laminates. J. Plast. Film Sheeting.

[58] Plastics Recyclers Europe (2020). Flexible Films Market in Europe—State of Play: Production, Collection and Recycling Data. https://www.plasticsrecyclers.eu/publications/.

[59] Industrial Packaging The Complete Guide to Shrink Film. https://www.industrialpackaging.com/hubfs/IP-Pillar-Guide-to-Shrink-Fim.pdf.

[60] Industrial Packaging The Complete Guide to Stretch Film for Shipping Protectionq. https://www.industrialpackaging.com/complete-guide-to-stretch-film.

