# Supplementary material for: Systematic Characterization and Property Enhancement of Recycled PE Flexible Film Packaging
Source: Polymers (Basel). 2025 Sep 13;17(18):2475. doi: 10.3390/polym17182475 (PMC12473179; doi:10.3390/polym17182475)
Supplement: Supplementary file 1 [file polymers-17-02475-s001.zip › polymers-3774021-supplementary.pdf]

polymers

MDPI

Article

Systematic Characterization and Property Enhancement of Recycled PE Flexible Film Packaging

Johanna Langwieser [1,2]*, Parvin Naderi [2] and Joerg Fischer [2,3]

[1] Competence Center CHASE GmbH, Altenberger Strasse 69, 4040 Linz, Austria
[2] Johannes Kepler University Linz, Institute of Polymeric Materials and Testing, Altenberger Strasse 69, 4040 Linz, Austria
[3] Johannes Kepler University Linz, Linz Institute of Technology (LIT) Factory, Altenberger Strasse 69, 4040 Linz, Austria
* Correspondence: johanna.langwieser@chasecenter.at

Academic Editors: Ngoc Nguyen, Ian Wyman

Received: 7 July 2025
Revised: 7 September 2025
Accepted: 10 September 2025
Published: 13 September 2025

Citation: To be added by editorial staff during production.

Copyright: © 2025 by the authors. Submitted for possible open access publication under the terms and conditions of the Creative Commons Attribution (CC BY) license (https://creativecommons.org/licenses/by/4.0/).

1. Tensile stress-strain curves

vPE-LD

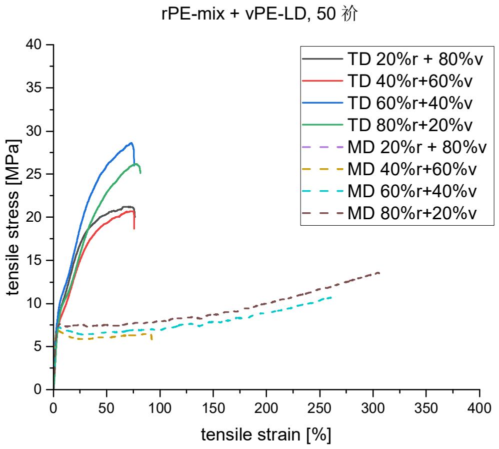

a)

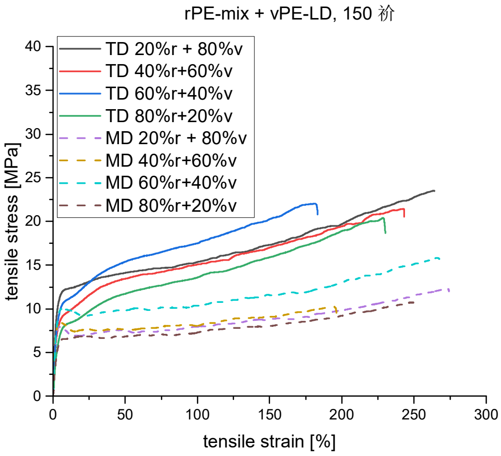

b)

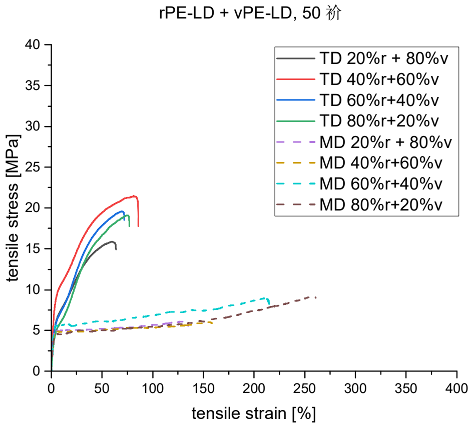

c)

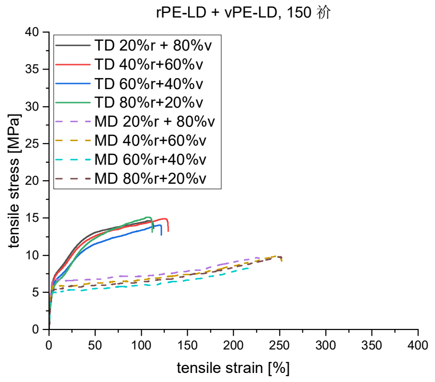

d)

Figure S1. Tensile stress-strain curves of a) rPE-mix + vPE-LD mixtures of 50 μm thickness in TD and MD, b) rPE-mix + vPE-LD mixtures of 150 μm thickness in TD and MD, c) rPE-LD + vPE-LD mixtures of 50 μm thickness in TD and MD, and d) rPE-LD + vPE-LD mixtures of 150 μm thickness in TD and MD.

vPE-LLD

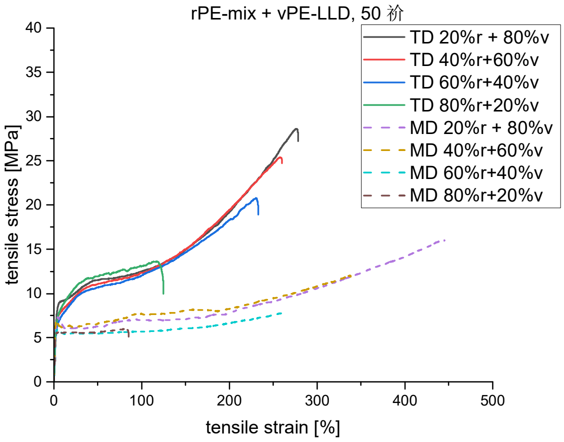

a)

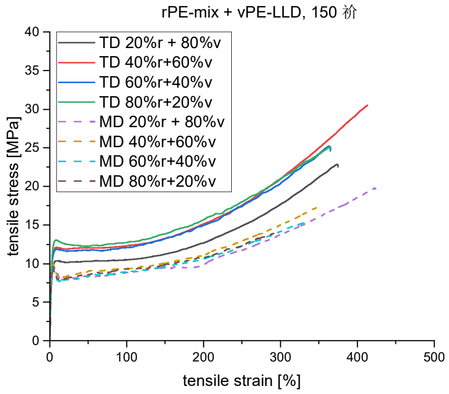

b)

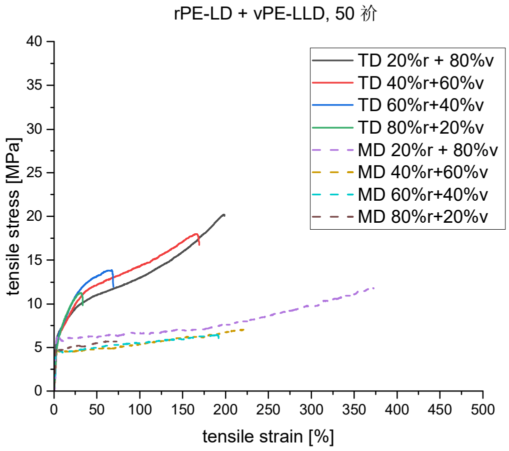

c)

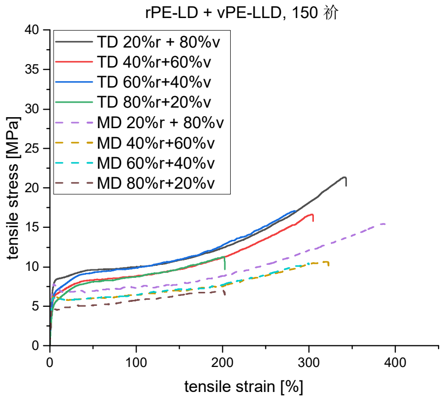

d)

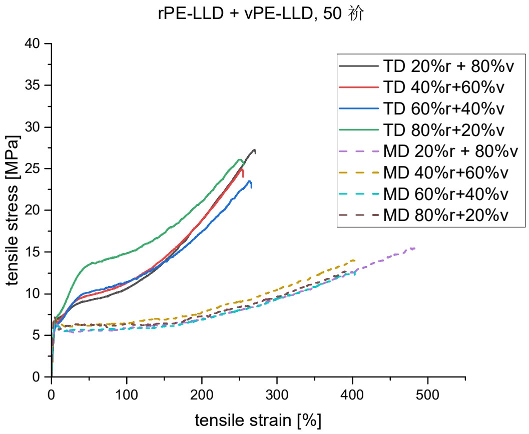

e)

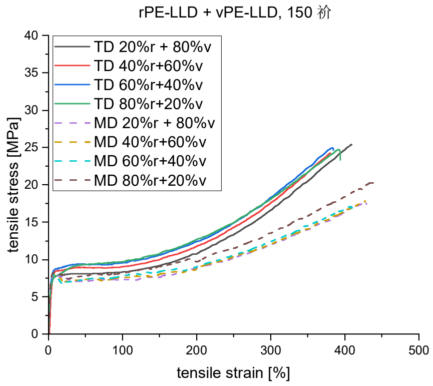

f)

Figure S2. Tensile stress-strain curves of a) rPE-mix + vPE-LLD mixtures of 50 μm thickness in TD and MD, b) rPE-mix + vPE-LLD mixtures of 150 μm thickness in TD and MD, c) rPE-LD + vPE-LLD mixtures of 50 μm thickness in TD and MD, d) rPE-LD + vPE-LLD mixtures of 150 μm thickness in TD and MD, e) rPE-LLD + vPE-LLD mixtures of 50 μm thickness in TD and MD, and f) rPE-LLD + vPE-LLD mixtures of 150 μm thickness in TD and MD.

Commercial

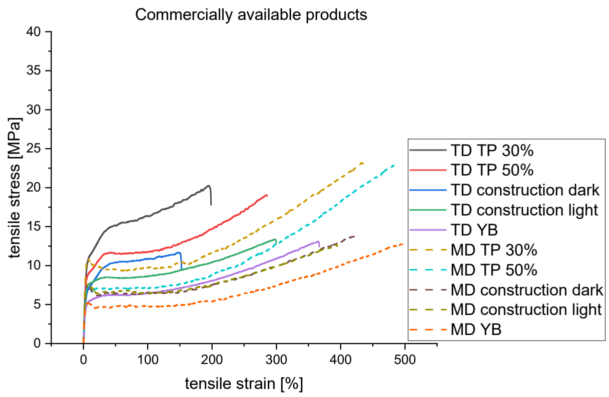

a)

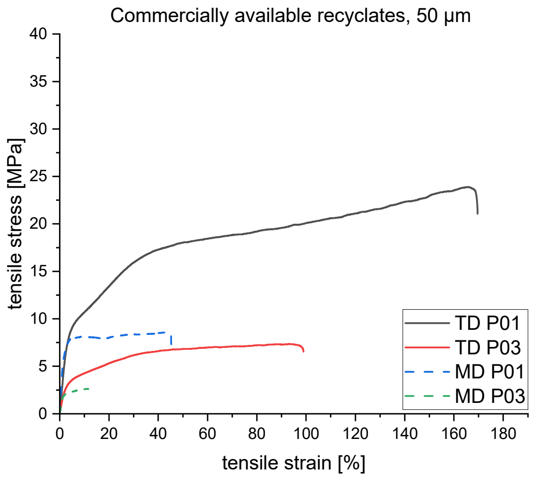

b)

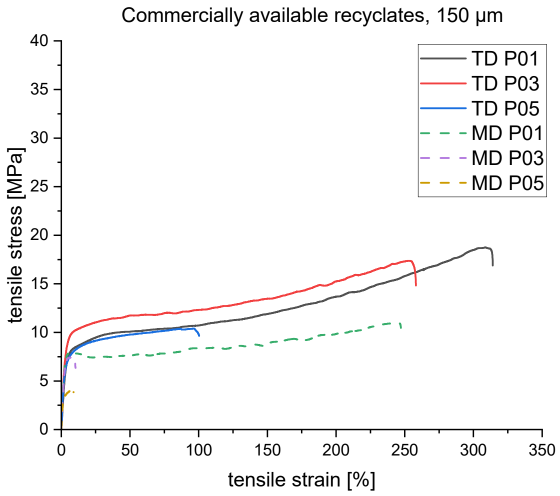

c)

Figure S3. Tensile stress-strain curves of a) of the commercially available products in TD and MD, b) commercially available recyclates of 50 µm in TD and MD, and c) commercially available recyclates of 150 µm in TD and MD.

2. Degrees of crystallinity

Table S1. Degrees of crystallinities of the lab-mixtures.

		vPE-LD	vPE-LLD
rPE-mix	100%v	26.5%	53.1%
	20%r + 80%v	14.5%	31.8%
	40%r + 60%v	18.0%	50.6%
	60%r + 40%v	14.4%	30.2%
	80%r + 20%v	13.5%	24.3%
	100%r	46.2%	
rPE-LD	20%r + 80%v	22.6%	43.9%
	40%r + 60%v	15.7%	48.9%
	60%r + 40%v	21.5%	42.8%
	80%r + 20%v	17.6%	43.3%
	100%r	43.2%	
rPE-LLD	20%r + 80%v		41.3%
	40%r + 60%v		46.4%
	60%r + 40%v		38.6%
	80%r + 20%v		35.8%
	100%r		44.5%

Table S2. Degrees of crystallinities of the commercially available products and the commercially available recyclates.

Commercially available products		Commercially available recyclates	
**TP 30%**	9.0%	**P01**	18.9%
**TP 50%**	9.9%	**P03**	13.9%
**Construction film dark**	7.0%	**P05**	12.7%
**Construction film light**	6.1%		
**YB**	4.0%		

3. DTA curves

vPE-LD

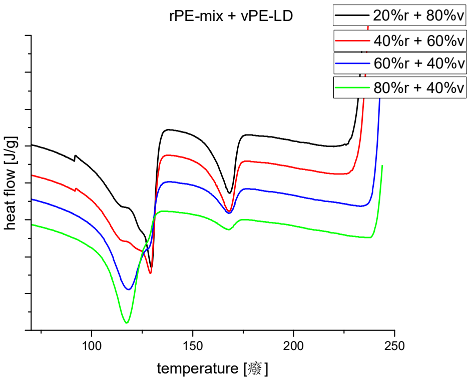

a)

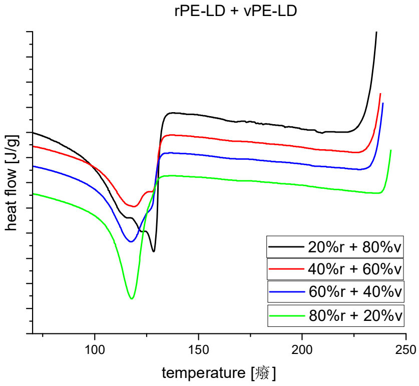

b)

Figure S4. Heat flow curves of a) rPE-mix + vPE-LD and b) rPE-LD + vPE-LD.

vPE-LLD

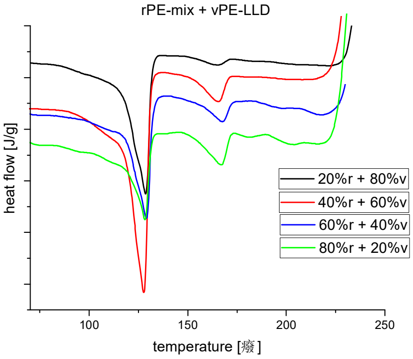

a)

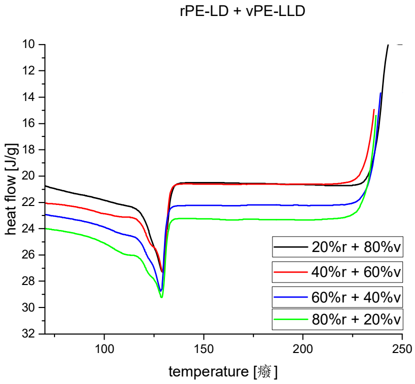

b)

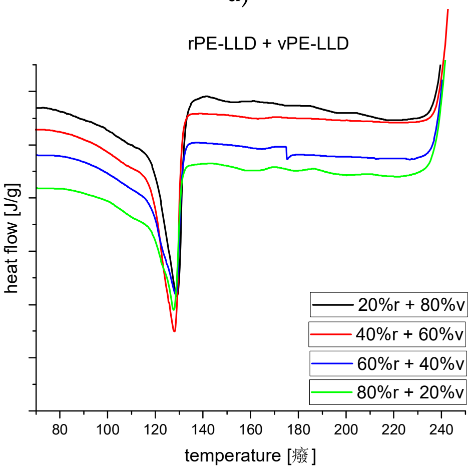

c)

Figure S5. Heat flow curves of a) rPE-mix + vPE-LLD, b) rPE-LD + vPE-LLD, and c) rPE-LLD + vPE-LLD.

Commercial

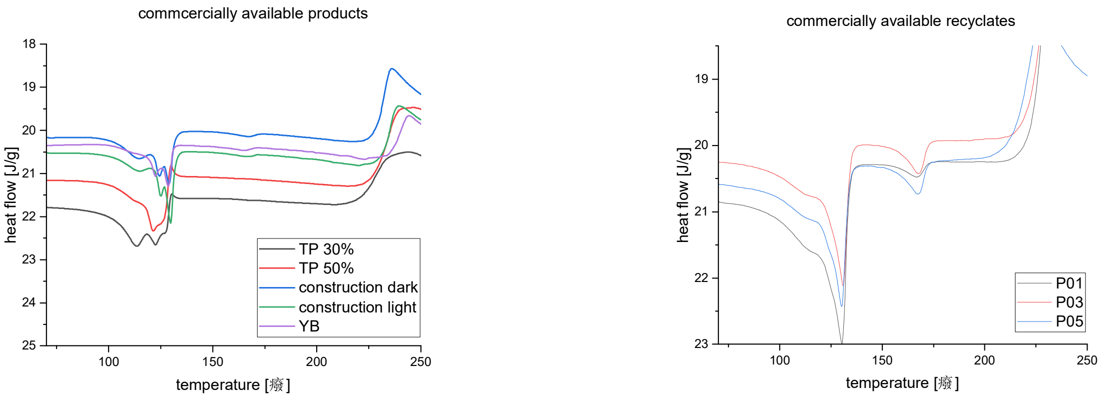

a) b)

Figure S6. Heat flow curves of a) the commercially available products and b) the commercially available recyclates.

4. UV-VIS spectra

vPE-LD

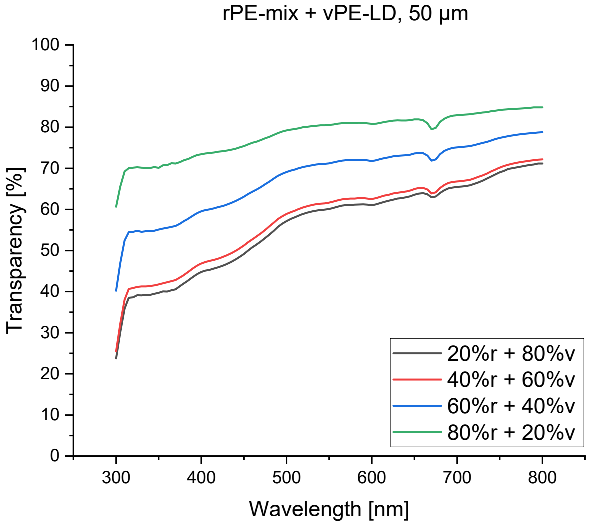

a)

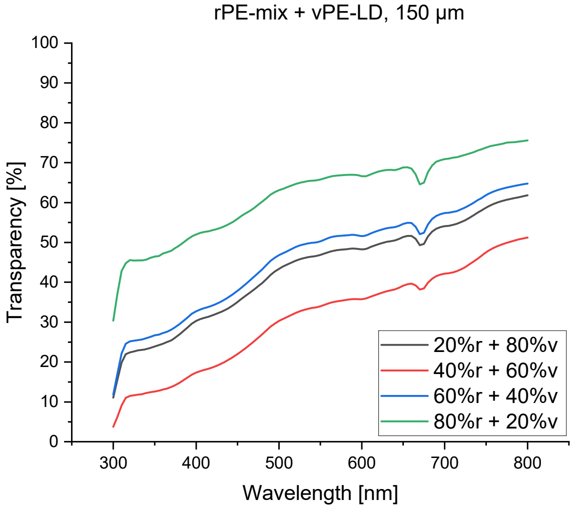

b)

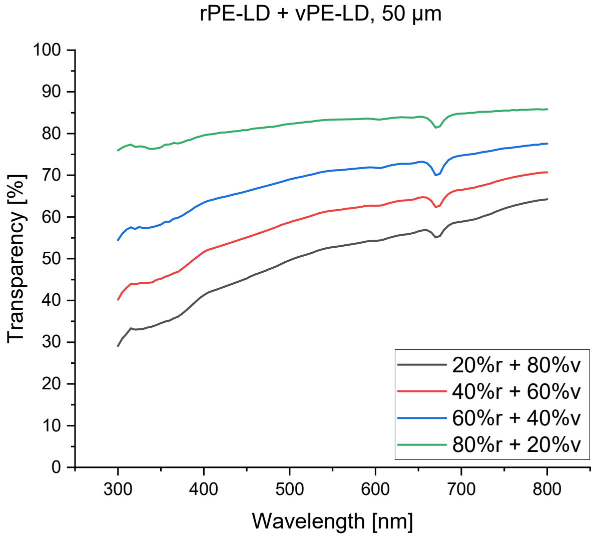

c)

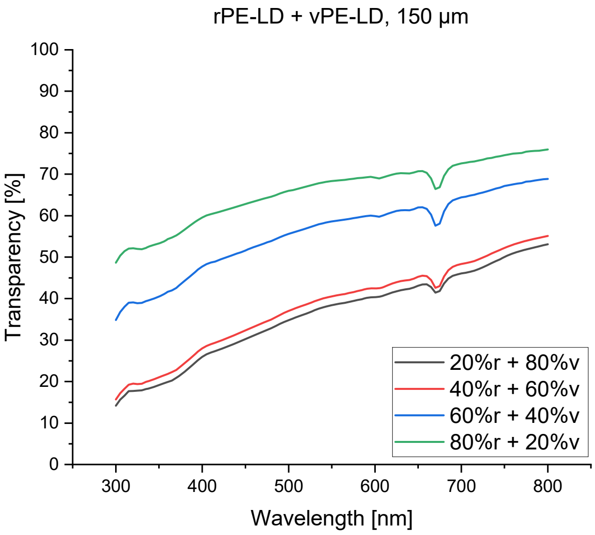

d)

Figure S7. UV-Vis spectra of a) rPE-mix + vPE-LD of 50 µm, b) rPE-mix + vPE-LD of 150 µm, c) rPE-LD + vPE-LD of 50 µm, and d) rPE-LD + vPE-LD of 150 µm.

vPE-LLD

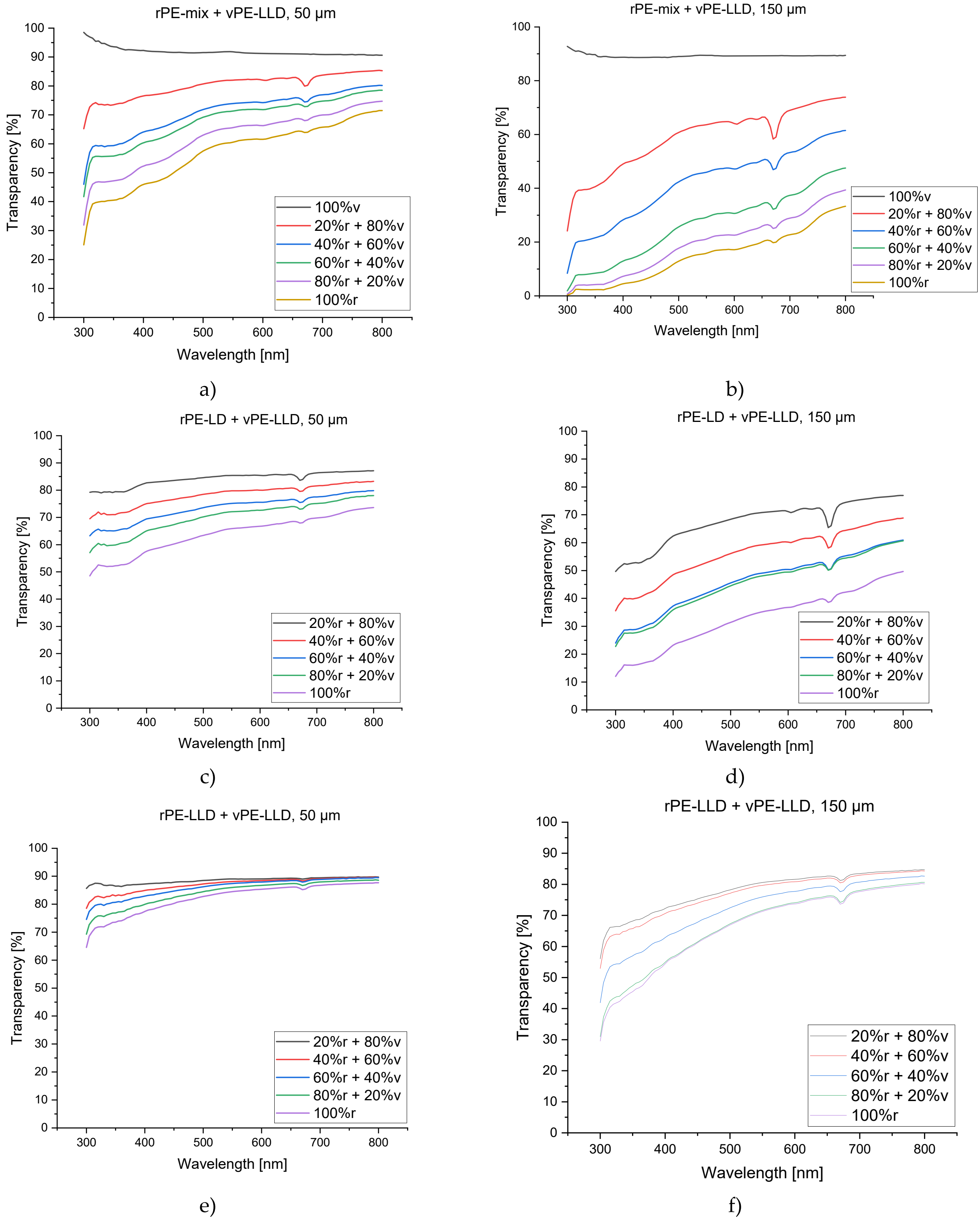

Figure S8. UV-Vis spectra of a) rPE-mix + vPE-LLD of 50 µm, b) rPE-mix + vPE-LLD of 150 µm, c) rPE-LD + vPE-LLD of 50 µm, d) rPE-LD + vPE-LLD of 150 µm, e) rPE-LLD + vPE-LLD of 50 µm, and f) rPE-LLD + vPE-LLD of 150 µm.

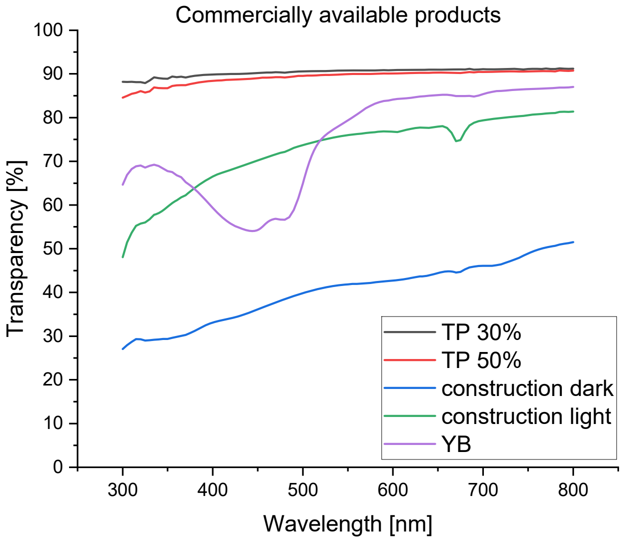

a)

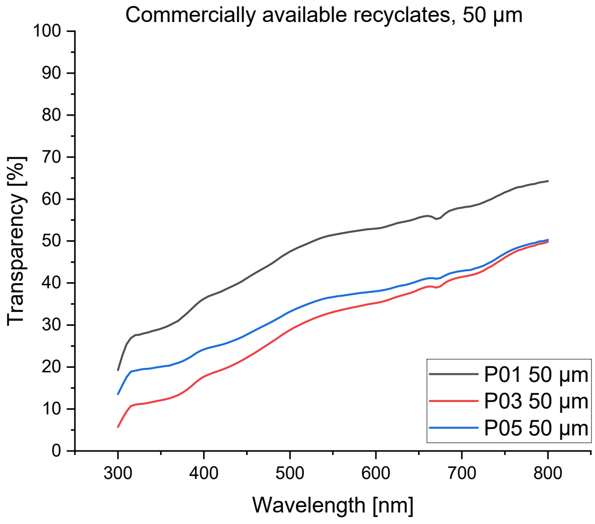

b)

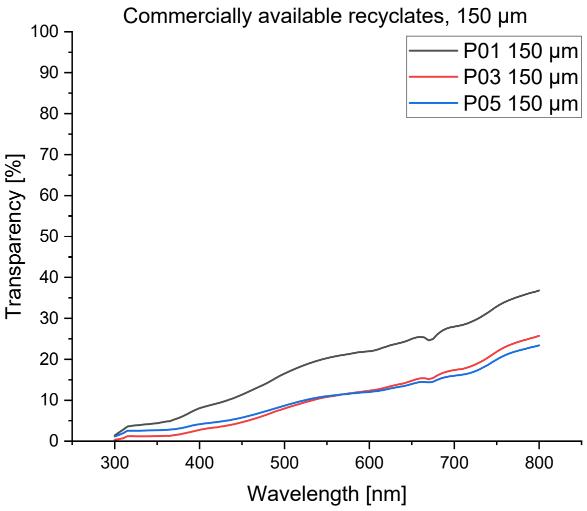

c)

Figure S9. UV-Vis spectra of a) the commercially available products, b) the commercially available recyclates of 50 µm, and c) the commercially available recyclates of 150 µm.

5. Properties of commercial recyclates and commercial products

Table S3. Properties of the commercially available products and the commercially available recyclates.

Commercially available products	OOT [°C]	Transparency [%]	Tensile TD		Tensile MD		Puncture	
			σ_b [Mpa]	ε_b [%]	σ_b [Mpa]	ε_b [%]	F [N]	L [mm]
**TP 30%**	225.3	90.4	22.3	419.0	18.6	191.6	1.2	7.3
**TP 50%**	220.9	89.5	22.9	474.8	18.1	283.2	1.0	7.3
**Construction film dark**	224.4	40.2	13.3	415.8	9.7	152.1	1.3	7.9
**Construction film light**	225.2	72.6	13.0	380.6	11.2	276.4	1.4	2.7
**YB**	231.5	74.6	12.0	468.3	11.9	360.9	0.8	7.9

Commercially available recyclates	OOT [°C]	Transparency [%]	Tensile TD		Tensile MD		Puncture	
		50 μm						
			σ_b [Mpa]	ε_b [%]	σ_b [Mpa]	ε_b [%]	F [N]	L [mm]
**P-01**	215.3	47.6	7.1	103.9	20.6	161.0	1.9	5.6
**P-03**	215.1	30.3	0.8	12.8	5.2	120.5	1.6	4.7
**P-05**	207.6	34.3					0.8	3.4
		150 μm						
			σ_b [Mpa]	ε_b [%]	σ_b [Mpa]	ε_b [%]	F [N]	L [mm]
**P-01**		18.7	7.7	188.5	17.3	300.4	3.1	4.5
**P-03**		10.6	2.5	38.9	15.9	264.3	3.2	4.4
**P-05**		10.6	1.9	16.1	8.7	113.8	2.6	4.0
